# RDTs as a source of DNA to study *Plasmodium falciparum* drug resistance in isolates from Senegal and the Comoros Islands

**DOI:** 10.1186/s12936-015-0861-6

**Published:** 2015-09-29

**Authors:** Nasserdine Papa Mze, Yaye Die Ndiaye, Cyrille K. Diedhiou, Silai Rahamatou, Baba Dieye, Rachel F. Daniels, Elizabeth J. Hamilton, Mouhamadou Diallo, Amy K. Bei, Dyann F. Wirth, Souleymane Mboup, Sarah K. Volkman, Ambroise D. Ahouidi, Daouda Ndiaye

**Affiliations:** Laboratory of Bacteriology-Virology, Hospital Aristide Le Dantec, 7325 Dakar, Senegal; Laboratoire of Parasitology and Mycology, Faculty of Medicine and Pharmacy, Cheikh Anta Diop University, 5005 Dakar, Senegal; Laboratory of National Malaria Control Programme, Moroni, Comoros; Department of Immunology and Infectious Diseases, Harvard TH Chan School of Public Health, Boston, MA 02115 USA; Department of Human Evolutionary Biology, Harvard University, Cambridge, MA USA; Broad Institute: The Broad Institute of MIT and Harvard, Cambridge, MA 02142 USA; School of Nursing and Health Sciences, Simmons College, Boston, MA 02115 USA

**Keywords:** *Plasmodium falciparum*, RDT, DNA source, *dhfr*, *dhps*, Comoros, Senegal

## Abstract

**Background:**

The World Health Organization has recommended rapid diagnostic tests (RDTs) for use in the diagnosis of suspected malaria cases. In addition to providing quick and accurate detection of *Plasmodium* parasite proteins in the blood, these tests can be used as sources of DNA for further genetic studies. As sulfadoxine-pyrimethamine is used currently for intermittent presumptive treatment of pregnant women in both Senegal and in the Comoros Islands, resistance mutations in the *dhfr* and *dhps* genes were investigated using DNA extracted from RDTs.

**Methods:**

The proximal portion of the nitrocellulose membrane of discarded RDTs was used for DNA extraction. This genomic DNA was amplified using HRM to genotype the molecular markers involved in resistance to sulfadoxine-pyrimethamine: *dhfr* (51, 59, 108, and 164) and *dhps* (436, 437, 540, 581, and 613). Additionally, the *msp1* and *msp2* genes were amplified to determine the average clonality between Grande-Comore (Comoros) and Thiès (Senegal).

**Results:**

A total of 201 samples were successfully genotyped at all codons by HRM; whereas, in 200 *msp1* and *msp2* genes were successfully amplified and genotyped by nested PCR. A high prevalence of resistance mutations were observed in the *dhfr* gene at codons 51, 59, and 108 as well as in the *dhps* gene at codons 437 and 436. A novel mutant in *dhps* at codon positions 436Y/437A was observed. The *dhfr* I164L codon and *dhps* K540 and *dhps* A581G codons had 100 % wild type alleles in all samples.

**Conclusion:**

The utility of field-collected RDTs was validated as a source of DNA for genetic studies interrogating frequencies of drug resistance mutations, using two different molecular methods (PCR and High Resolution Melting). RDTs should not be discarded after use as they can be a valuable source of DNA for genetic and epidemiological studies in sites where filter paper or venous blood collected samples are nonexistent.

**Electronic supplementary material:**

The online version of this article (doi:10.1186/s12936-015-0861-6) contains supplementary material, which is available to authorized users.

## Background

Malaria remains a major public health problem as it is responsible for 207 million cases and 627,000 deaths worldwide [1]. Currently, 90 % of the deaths attributable to this infection occur in sub-Saharan Africa. To combat this disease, many programmes have implemented control measures such as the distribution of long-lasting insecticide-treated mosquito nets, the use of artemisinin-based combination therapy (ACT) for the treatment of uncomplicated *Plasmodium falciparum* malaria, and the introduction of rapid diagnostics tests (RDTs) in health facilities for malaria diagnosis. Such interventions have contributed to a dramatic drop in malaria-attributable deaths worldwide [1].

Widespread RDT use has greatly assisted in the accurate diagnosis of presumed malaria cases in regions where microscopy (or molecular methods) are non-existent. RDTs are based on antigen detection and these antigens, as well as parasite DNA, remain on the discarded RDT after it has been used for patient diagnosis. The widespread use of RDTs for diagnosis creates a valuable resource for population-based studies as RDTs have been shown to be suitable material for genetic studies, as DNA can be extracted from RDTs [2–5].

To validate the utility of field-collected RDTs for typing of drug resistance markers, two markers involved in sulfadoxine-pyrimethamine (SP, Fansidar^®^) resistance were selected: *dhfr* and *dhps* genes, as these both drugs are routinely used in the populations of interest (Senegal and Comoros). Until recently, SP was one of the main molecules for the treatment of uncomplicated malaria [6, 7]. However, resistance to SP has evolved quickly [8]. SP is a combination of two drugs that act at two successive stages of the parasite folate pathway. Sulfadoxine inhibits the *dihydropteroatesynthetase* gene (*dhps*), while pyrimethamine inhibits *dihydrofolatereductase* gene (*dhfr*). Point mutations in the *dhfr* and *dhps* genes confer resistance to pyrimethamine and sulfadoxine, respectively, with a decrease of in vitro susceptibility of *P. falciparum* in relation to the number of mutations of each gene [9–13]. Although this molecule has seen a relative decline of its therapeutic efficacy, it is now used in Senegal for the Intermittent Preventive Treatment (IPT) of pregnant women and children against malaria, and in Comoros, for IPT of pregnant women, a policy change that was implemented in 2003 and 2004, respectively.

While many studies measuring the prevalence of *dhfr* and *dhps* mutations have been conducted previously in Senegal [14–19], few have been performed in Comoros [20–22], largely due to a lack of clinical studies in which venous blood or filter paper are collected to study parasite molecular markers. The objectives of this study were (1) to assess the feasibility of DNA extraction from field-collected RDTs, (2) to study gene mutations *dhfr* (S108N, N51I, C59R and I164L) and *dhps* (A437G, S436F, K540E, A581G and A613T/S) from samples of Senegal and Comoros with high-resolution melting technology and compare the prevalence between these two countries. *Msp* typing was used as a tool to compare the average multiplicity of infection between the two countries.

## Methods

### Study sites and sample collection

The Comoros islands are located on the southeastern coast of Africa in the Mozambique canal, and represent a country where malaria transmission is high [23]. In Grand-Comore, the largest of the four islands of Comoros, the malaria transmission is meso- to hyperendemic (EIR between 10 and 200) [20]. In contrast, Senegal has Sahalien characteristics marked by a transmission that is seasonal and short. Samples collected for this study were from Thiès, an urban area 70 km east of Dakar, where malaria is hypoendemic [24], with an EIR between 1 and 5.

In Grande-Comore, RDT samples were collected from 2012 to 2013 by the National Malaria Control Programme in the city of Moroni, where malaria is hypoendemic; and in two hospitals Mitsamiouli and Mbeni where malaria is mesoendemic and meso to hyperendemic respectively [20]. In Senegal, RDT samples were collected in 2010 at the Service de Lutte Anti-Parasitaire (SLAP) clinic, in Thiès. Malaria positive RDTs were stored at room temperature with desiccant at both sites.

### Rapid diagnostic tests and DNA extraction

In Senegal, SD BIOLINE Malaria rapid test for Pf alone (SD Bioline, 05FK50) was used while in Comoros two different tests were used: Malaria pLDH/HRP2 Combo (Access Bio, PBX-KM30003) and SD BIOLINE Malaria Ag Pf/Pan (SD Bioline, 05FK60). The proximal third of the nitrocellulose membrane was chosen for DNA extraction as previously described [2]. DNA was extracted with the QIAamp DNA Mini kit (Qiagen) according to the manufacturer’s recommendations for filter paper. In total, 204 samples (124 from Grande-Comore and 80 from Thiès) were extracted and genotyped.

### Single nucleotide polymorphism analyses by high resolution melting

SNP analysis for mutations in *dhfr* (S108N, N51I, C59R and I164L) and *dhps* (A437G, S436F, K540E, A581G and A613T/S) genes were conducted by High Resolution Melting (HRM) analysis using a Light Scanner LS-32, according to previously published methodology [18]. Asymmetric PCR was performed with final primer concentrations of 0.25 µM reverse primer; 0.05 µM forward primer; and a final probe concentration of 0.02 µM. The cycling and melting conditions for *dhps* amplification were as follows: 95 °C denaturation for 1 min, followed by 55 cycles of (95 °C for 5 s and 66 °C for 30 s) a pre-melt cycle of 5 s each at 95 °C and 37 °C, followed by a melt from 45 to 90 °C at a 0.30 °C/s. For the 437 *dhps* assay, cycling and melting conditions were as follows: 95 °C denaturation for 1 min, followed by 55 cycles of (95 °C for 5 s, 66 °C for 30 s, 74 °C for 30 s) a pre-melt cycle of 5 s each at 95 and 37 °C, followed by a melt from 45 to 90 °C at a 0.30 °C/s. The cycling and melting conditions for *dhfr* amplification were as follows: 95 °C denaturation for 1 min, followed by 55 cycles of (95 °C for 5 s, 56 °C for 30 s) a pre-melt cycle of 5 s each at 95 and 37 °C, followed by a melt from 45 to 90 °C at a 0.30 °C/s.

### DNA sequencing

PCR sequencing (Sanger sequencing) of the *dhps* 436/437 amplicon was performed by using the same primers used in the HRM reaction (Forward: GAATGTTTGAAATGATAAATGAAGGTGCTA and Reverse: CAGGAAACAGCTATGACGAAATAATTGTAATACAGG TACTACTAAATCTCT). Sequencing was performed in both the forward and the reverse direction by Macrogen and contigs were assembled using Lasergene 10.

### Allelic typing of *Plasmodium falciparum msp1* and *msp2*

The polymorphic regions (block 2 of *MSP1* and block 3 of *MSP2*) were amplified as previously described [25]. All PCR reactions were carried out in a total volume of 20 µl containing 6 µl Gotaq, 0.5 µM of each primer, and 11 µl reagent grade water. In the first round reaction (nest 1), 1 µl of genomic DNA was added as a template. In the second nested reaction (nest 2), 1 µl of the nest 1 PCR product was used as DNA template.

The cycling conditions for the nest 1 PCR were as follows: initial denaturation at 95 °C for 5 min, followed by 35 cycles of (94 °C for 1 min, 58 °C for 2 min, 72 °C for 2 min), with a final extension cycle of 72 °C for 3 min. The cycling conditions for the nest 2 PCR were as follows: initial denaturation at 95 °C for 5 min, followed by 35 cycles of (94 °C for 1 min, 61 °C for 2 min, 72 °C for 2 min), with a final extension cycle of 72 °C for 3 min. Positive controls (3D7 for K1 and IC27 alleles, Dd2 for MAD20 and FC27 alleles, and 7G8 for the RO33 allele) and negative control (reagent grade water alone) were run in each PCR reaction. PCR products were analyzed on 2 % agarose gels stained with ethidium bromide and visualized by UV trans-illumination (BioradGel Doc™ XR + System with Image Lab). The size of resulting DNA bands were approximated using Gene Ruler 100 bp DNA ladder marker (Quick Load).

### Statistical analyses

Statistical analyses were performed using the Z-test for two population proportions to compare mutant allele prevalence between Comoros and Senegal samples. For all tests, the significance level was α = 0.05.

## Results

### The prevalence of mutations in *dhfr* and *dhps*

Genotyping of *dhps* and *dhfr* genes was performed for 201 samples (n = 124 for Grande-Comore and n = 77 for Thiès). For *dhfr* codons 51/59, only 62 samples from Thiès and 96 samples from Grande-Comore yielded a successful genotyping result; whereas at all other loci, genotyping was successful for all samples (Table 1).

**Table 1:** Utility of RDTs as a source of DNA for genotyping loci involved in diversity and drug resistance

RDT Brand	Number RDT	HRM^a^	HRM^a^	PCR^b^	PCR^b^	Fragment used RDT
		Positive	Negative	Positive	Negative	
SD Bioline malaria Rapid test pf	80	77	3	77	3	1/3 NC
SD Bioline malaria Ag Pf/Pan	13	13	0	13	0	1/3 NC
Malaria pLDH/HRP2 combo	111	111	0	110	1	1/3 NC
Total	204	201^c^	3	200	4	–

^a^The HRM method was used for genotyping *dhfr* and *dhps* genes

^b^PCR was used to genotype *msp1* and *msp2*

^c^For codons 51/59, only 62 samples from Thiès and 96 samples from Grande-Comore gave a comprehensive genotyping

**Fig. 1:**
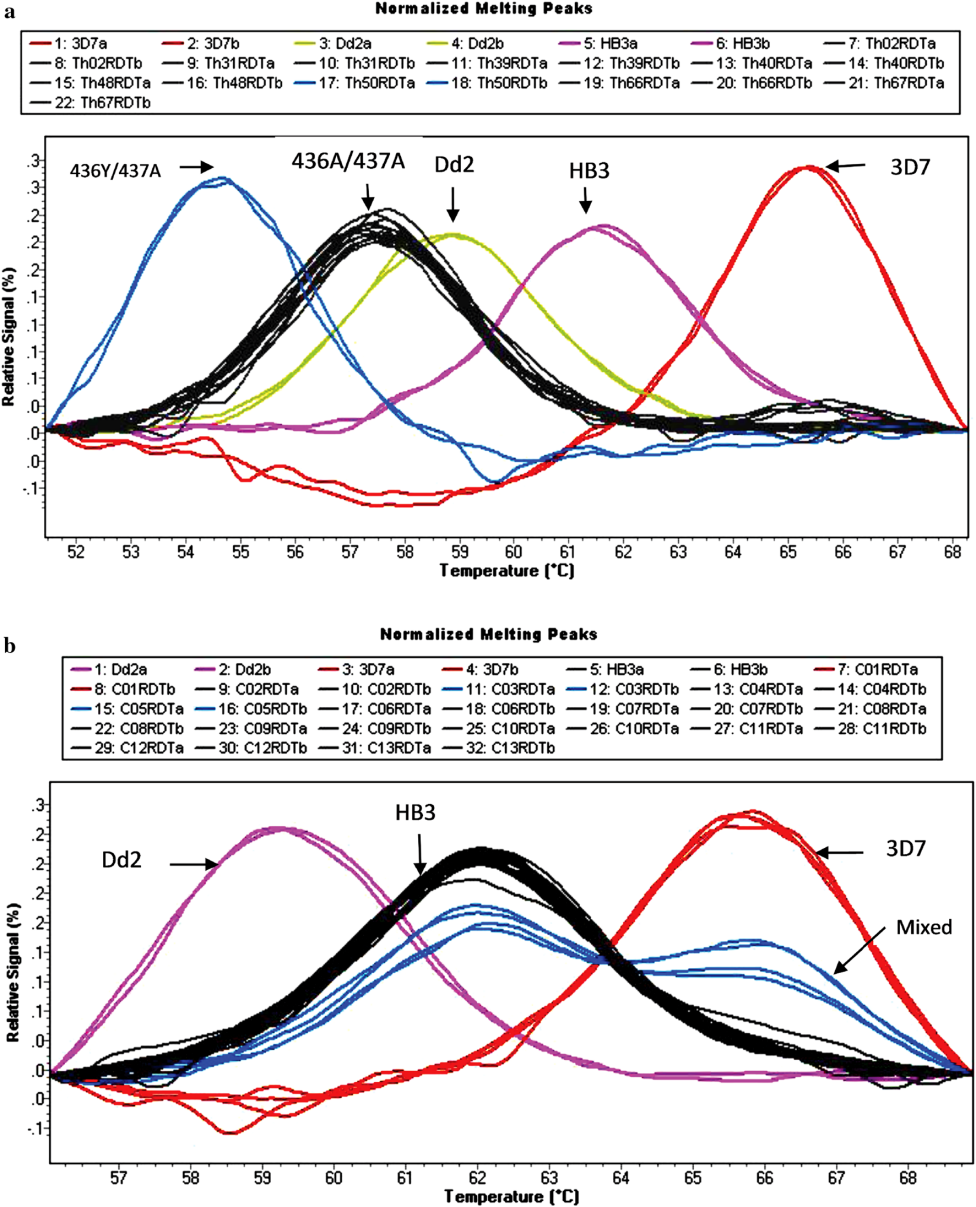
HRM peak profiles for wild-type and mutant *dhps* 436/437 alleles. **a** The *3D7 peak* represents the profile for the wild-type at codon 436 and mutant profile for the 437 codon. The *Dd2 peak* presents the profile for the two mutant Codons 436/437. The *HB3 peak* presents the wild-type profile of the two codons. The *436A/437A* *peaks*, represented the mutations found by Daniels et al. in Senegal [18]. The *blue peak* from sample Th50 RDT represents a new mutant allele. **b** The peaks C03 and C05 represent mixed samples (mutant + wild). The Dd2 peak presents the mutant profile for both codons 436 and 437. The *HB3 peak* represents the wild-type profile for both codons 436 and 437. The *3D7 peak* represents the profile for the wild-type at codon 436 and mutant profile for the 437 codon

**Fig. 2:**
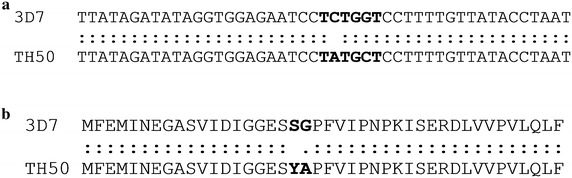
Sequencing sample Th050. **a** DNA; **b** amino acid. To confirm the new mutant profile obtained from HRM, sequencing was performed. After alignment of the nucleotides sequences we found new mutations in *dhps* 436Y/437A. The TAT codon corresponds to tyrosine (Y) and the codon GCT corresponding to the alanine (A)

In Thiès, for the *dhfr* gene, 90 % (54/60) of the samples had the mutant allele at codons *dhfr* N51C/C59R, and 95.8 % (68/71) had the mutant allele at codon *dhfr* S108 N. For the *dhps* gene 1.33 % (1/75) had the A613T mutant allele and 53.9 % (41/76) of the samples had the A437G mutant allele. At the S436 codon of *dhps* 12 % (9/77) of the samples had the mutant allele S436F, a recently characterized mutation described previously [18]. For all other codons (namely, *dhfr* I164, *dhps* K540, and *dhps* A581) 100 % of the samples had the wild-type allele. Previously unreported mutations were observed for *dhps* gene at codons 436/437 by HRM (Fig. 1a) and were confirmed to be S436Y/437A by Sanger sequencing (Fig. 2).

In Grande-Comore, for the *dhfr* gene, 93.4 % (115/123) had the S108 N mutation, 62.5 % (60/96) of the samples had the N51I mutant allele, and 69.8 % (67/96) of all samples had the C59R mutant allele. For the *dhps* gene, at position A437, 29.8 % (37/124) of samples had the mutant allele A437G. For all other codons (namely, *dhfr* I164, *dhps* K540, *dhps* A581, *dhps* A613, and *dhps* S436), 100 % of the samples had the wild-type allele.

A statistically significant difference was observed in the prevalence of mutations between the Comoros and Senegal for the *dhfr* gene codons 51 (0.0001), and 59 (p = 0.003), and *dhps* gene codons 436 (p = 0.0001) and 437 (p = 0.0007). The prevalence of mutations on the *dhfr* gene at codon 51 and 59 and *dhps* gene at codon 436 and at codon 437 were higher in Thiès compared to Grande-Comore.

**Fig. 3:**
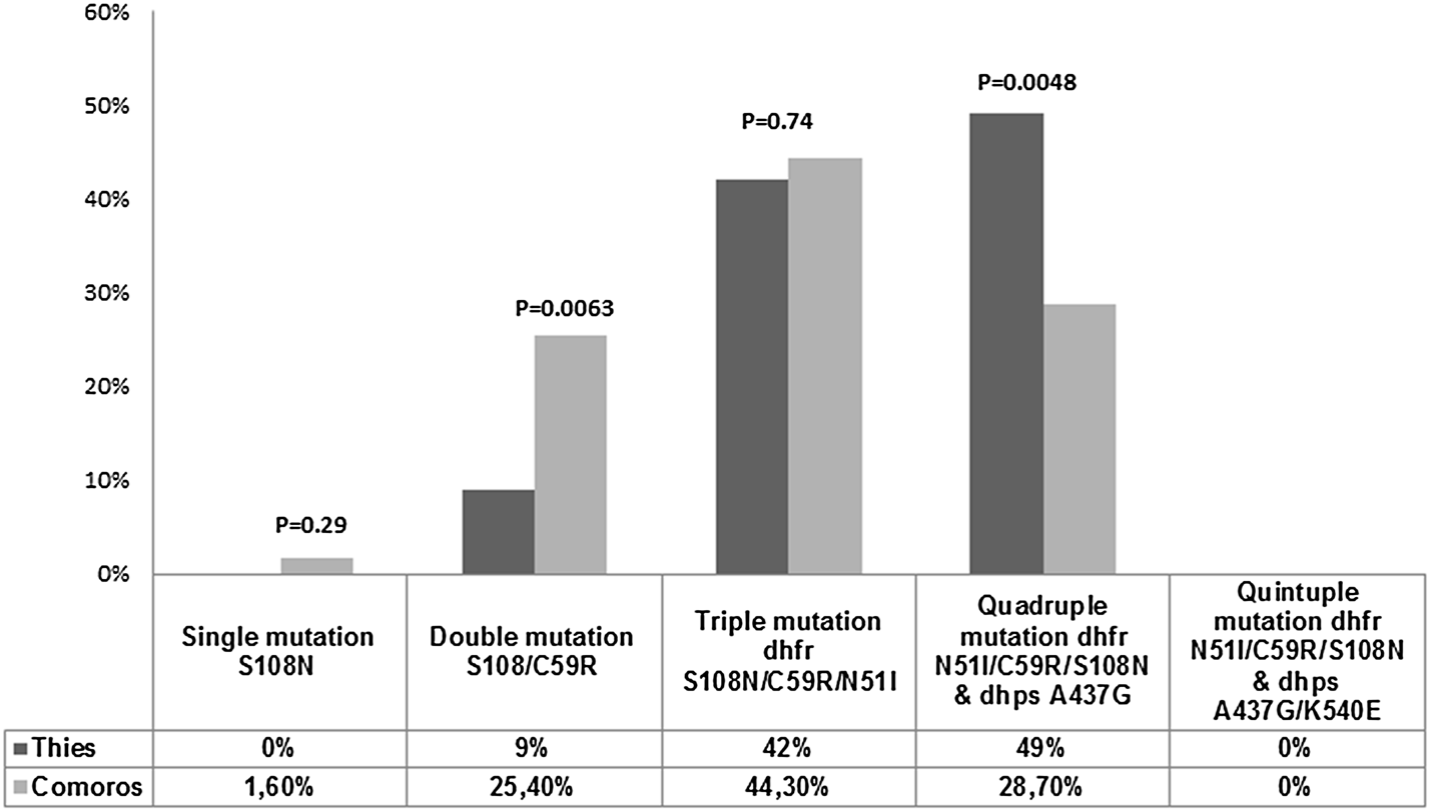
Prevalence of single, double, triple, quadruple and quintuple mutation, in Comoros and Senegal. The prevalence of single (S108N), double (S108N/C59R), triple (N51I/C59R/S108N), quadruple (N51I/C59R/S108N/A437G) and quintuple (N51I/C59R/S108N/A437G/K540E) mutation was determined by counting the number of mutants present only in one, two, three, four or five Codons respectively. The Z-test for two population proportions is used to determine the P values, with a significance level of α = 0.05

The single mutation (S108N alone, in the absence of other *dhfr* mutations) was not found in Thiès; whereas, in Grande-Comore it was present at 1.6 % (2/122) (Fig. 3). The prevalence of the double mutation (*dhfr* C59R and S108N) was 9 % (6/67) in Thiès and 25.4 % (31/122) in Grande-Comore (Fig. 3). However, the triple mutation (*dhfr* S108N, N51I, and C59R) was present at similar prevalences in both countries with 44.3 % (28/122) in Grande-Comore and 42 % (28/67) in Thiès; whereas the quadruple mutation (*dhfr* S108N, N51I, C59R, and *dhps* A437G) was observed at 28.7 % (35/122) in Grande-Comore and 49 % (33/67) in Thiès (Fig. 3). No quintuple mutations (*dhfr* S108N, N51I, C59R, *dhps* A437G, and K540E) were observed in either country. Statistically, there was no significant difference in the prevalence of single (p = 0.29) and triple mutation (p = 0.74); however there was significance difference in the prevalence of double mutation (p = 0.0063) and the quadruple mutation (p = 0.0048) between the two countries using Z-test for 2 population proportions (Fig. 3).

When comparing the number of mixed alleles (mutant + wild) detected by HRM, overall mixed alleles were more prevalent in Grande-Comore samples than in samples from Thiès. An example of the HRM output for mixed alleles is shown in Fig. 1b. The difference in mixed alleles at *dhfr* position 108 and *dhps* position 437 was not significantly different between the two sites [*dhfr* 108: Grande-Comore 4 mixed samples (3 %); Thiès 3 mixed (4 %) samples (p = 0.77); *dhps* 437: Grande-Comore 5 mixed samples (4 %); Thiès 2 mixed samples (2.6 %) (p = 0.60)]. In contrast, in Grande-Comore a significant difference in the prevalence of mixed alleles at *dhfr* 51/59 was observed. In Grande-Comore, 27 mixed samples (28 %) at *dhfr* codons 51/59 compared to six mixed samples (9 %) in Thiès (p = 0.003) were observed.

### Genotyping using *msp1* and *msp2*

Having validated the RDT-extracted DNA to study markers of drug resistance, the average clonality in both populations was determined by *msp* typing. With samples from Thiès, a total of 77 samples were positive for *msp1* and *msp2* genes, with 3 samples failing to amplify (Table 1). In total, 36 samples (64.2 %) contained polyclonal infection at least with 2 clones. The average multiplicity of infection (MOI) for all isolates from Thiès was estimated to be 1.57.

With samples from Grande-Comore, a total of 123 samples were positive for *msp1* and *msp2* genotyping, with only a single sample failing amplification (Table 1). In total, 42 samples (43.7 %) had polyclonal infections at least with 2 clones. The average multiplicity of infection (MOI) was 1.47.

## Discussion

The WHO recommends that all suspected malaria cases be confirmed by RDTs. RDTs facilitate the diagnosis of malaria by providing evidence of the presence of specific *Plasmodium* antigens in human blood without the need for electricity or advanced microscopy training. Previous studies have shown that it is possible to extract *Plasmodium* DNA from RDTs [2, 3, 5]. This study sought to address whether the use of stored, discarded RDTs from field sites can serve as source of DNA for genomic studies in populations in which whole blood or filter paper-based DNA samples are unavailable.

There are many factors that could affect the efficacy of extracting DNA from field-based RDTs. Some of these factors include variable preparation of RDTs (variable blood volumes), and storage conditions. Storage conditions in field sites can differ, introducing the possibility of bacterial or fungal contamination as well as DNA degradation. Another variable to consider is the proportion of human DNA to *Plasmodium* DNA on each RDT as the ability to detect *Plasmodium* genes will depend on the specific concentration of *Plasmodium* DNA. Taking all these field-based realities into consideration, the methodology outlined by Cnops et al. [2] was applied to the field as overall, RDTs represent a cost-efficient method for the conservation, transport, and storage of DNA samples. This study addresses the reality of using RDTs from malaria endemic sites as sources of genetic material for performing molecular studies.

To test the performance of field-RDT extracted DNA for genetic experiments, two genetic methodologies were employed: high resolution melting (HRM) for drug resistance markers (*dhfr* and *dhps*) and *msp* typing by PCR to determine clonality. Differences in malaria transmission reported between Grande-Comore (meso-hyperendemic) and Thiès (hypoendemic) were compared by *msp* typing, and a small, but insignificant difference was observed in the multiplicity of infection for *msp* genes (*msp1* and *msp2*) (MOI = 1.57 in Thiès and MOI = 1.47 in Grande-Comore). This small difference is interesting given the predicted higher endemicity of Grande-Comore compared to Thiès.

HRM is a method that uses post-PCR melting analysis used to identify SNPs in nucleic acid sequences, and has a limit of detection of 100 fg of DNA [18]. In this study, successful genotyping by HRM was performed from DNA extracted from RDTs. An additional methodological advantage of HRM is that known mutations can be classified and novel mutations can be identified, such as the 436Y/437A mutations in *dhps*, which were identified in a sample from Thiès (Fig. 1a). When results are compared from RDT-extracted DNA to previous results obtained with the filter-paper extracted DNA using the same HRM method from the same population (Thiès, Senegal), similar frequencies for *dhfr* mutations at codons 51, 59 and 108 and *dhps* mutations at codon 437 [18] were observed.

Having confidence in the results obtained for Thiès, Senegal, this validated approach was applied to a population lacking filter paper or venous blood DNA samples: Comoros. In 2005, with declining CQ efficacy in Comoros, SP monotherapy efficacy studies were performed and while SP showed good clinical and parasitological responses (90–100 %), concerns were raised regarding the high prevalence of *dhfr* S108N mutations [21, 22]. In 2003, CQ was the first line treatment for malaria, with SP as the second-line drug. In 2005, CQ was replaced by artemether–lumefantrine for treatment of clinical malaria, although SP is still used for IPT in pregnant women to date. Very few molecular studies of drug resistance markers have been published for Comoros [20–22]. Interestingly, the number of mutations in Grande-Comore were significantly higher in this study (2012–2013) compared to what was shown by Rebaudet et al. [20] (Additional file 1: Table S1), specifically at *dhfr* codon 51 (38.5 % in 2010 compared to 62.5 % in 2012–2013; p = 0.02), *dhfr* position 108 (50 % in 2010 compared to 93 % in 2012–2013; p < 0.05) and *dhps* codon 437 (4 % in 2010 compared to 29.8 % in 2012–2013; p = 0.006). A significant increase in the number of mixed alleles at *dhfr* C59 and C59R (7.7 % in 2010 compared to 28 % in 2012–2013; p = 0.03) was observed and a trending increase in the number of mixed alleles at *dhps* A437 and A437G (Fig. 1b); albeit non-significant (0 % in 2010 compared to 4 % in 2012–2013, p = 0.3) (Additional file 1: Table S1). However; a decrease in mixed alleles *dhfr* S108 and S108 N (15.4 % in 2010 compared to 3.25 % in 2012–2013; p = 0.01) was observed. Of concern, a statistically significant increase in the triple mutation *dhfr* N51I/C59R/S108 N (50 % in 2010 compared to 89.6 % in 2012–2013; p < 0.05) was observed in this study. Other studies performed in Comoros in 2004–2006 [26] and 2006 [27] showed similar triple mutation frequencies (53 and 45 %, respectively) to the 50 % reported by Rebaudet et al. [20], indicating a very recent increase in triple mutation prevalence.

This high prevalence of mutations could potentially be explained by the use of SP for IPT but also by the fact that SP has been used as treatment for uncomplicated malaria [28]. On the other hand, the widespread use of trimethoprim-sulfamethoxazole (Cotrimoxazole^®^ or Bactrim^®^) in Comoros, a drug similar to SP that has the ability to select for resistant mutants [29, 30] could also potentially explain the higher frequencies of mutant alleles between 2010 and 2012/2013.

Drug resistance mutations highly associated with drug failure in both countries were compared, and encouragingly, the quintuple mutant *dhfr* 51I/59R/108N + *dhps* 437G/540E which is a key mutation associated with clinical failure to SP [31, 32] was not observed, in either Grande-Comore or in Thiès, Senegal. This lack of the quintuple mutation has been reported in previous studies [14–19], with the exception of one study by Andriantsoanirina et al. where a 0.4 % prevalence of the mutation A437G/K540E was observed in Comoros [27]. While, SP can be still used for ITP as recommended by World Health Organization [33], vigilance is needed as the WHO recommends replacing SP monotherapy if the prevalence of the K540E mutation exceeds 50 % in the population [33, 34]. These results taken together suggest that yearly monitoring of SP drug resistance in Comoros needs to be a policy priority as it will be important to assess the future efficacy of SP as a drug for IPT.

## Conclusion

In conclusion, drug resistance monitoring in Comoros is a critical area of future ongoing research. DNA extracted from RDTs can be a useful source of DNA for genetic epidemiological studies. DNA extracted from RDTs can be an alternative to filter paper or venous blood when such samples are not available.

## Additional file

Additional file 1:
**Table S1.** Prevalence of *dhfr* and *dhps* alleles in Comoros over time. Prevalences in *dhfr* and *dhps* mutations were compared from Rebaudet et al. in 2010 and this study in 2012–2013.

## References

[1] World Malaria Report 2013. World Health Organization. 2013. http://www.who.int/malaria/publications/world_malaria_report_2013/.

[2] Cnops L, Boderie M, Gillet P, Van Esbroeck M, Jacobs J (2011). Rapid diagnostic tests as a source of DNA for *Plasmodium* species-specific real-time PCR. Malar J.

[3] Ishengoma DS, Lwitiho S, Madebe RA, Nyagonde N, Persson O, Vestergaard LS (2011). Using rapid diagnostic tests as source of malaria parasite DNA for molecular analyses in the era of declining malaria prevalence. Malar J.

[4] Morris U, Aydin-Schmidt B, Shakely D, Martensson A, Jornhagen L, Ali AS (2013). Rapid diagnostic tests for molecular surveillance of *Plasmodium falciparum* malaria-assessment of DNA extraction methods and field applicability. Malar J.

[5] Veron V, Carme B (2006). Recovery and use of *Plasmodium* DNA from malaria rapid diagnostic tests. Am J Trop Med Hyg.

[6] Olliaro P, Nevill C, LeBras J, Ringwald P, Mussano P, Garner P (1996). Systematic review of amodiaquine treatment in uncomplicated malaria. Lancet.

[7] White NJ, Nosten F, Looareesuwan S, Watkins WM, Marsh K, Snow RW (1999). Averting a malaria disaster. Lancet.

[8] Yuthavong Y (2002). Basis for antifolate action and resistance in malaria. Microbes Infect.

[9] A-Elbasit IE, Khalil IF, Elbashir MI, Masuadi EM, Bygbjerg IC, Alifrangis M (2008). High frequency of *Plasmodium falciparum* CICNI/SGEAA and CVIET haplotypes without association with resistance to sulfadoxine/pyrimethamine and chloroquine combination in the Daraweesh area, in Sudan. Eur J Clin Microbiol Infect Dis.

[10] Das S, Chakraborty SP, Hati A, Roy S (2013). Malaria treatment failure with novel mutation in the *Plasmodium falciparum* dihydrofolatereductase (*pfdhfr*) gene in Kolkata, West Bengal, India. Int J Antimicrob Agents.

[11] Mombo-Ngoma G, Oyakhirome S, Ord R, Gabor JJ, Greutélaers KC, Profanter K (2011). High prevalence of *dhfr* triple mutant and correlation with high rates of sulphadoxine-pyrimethamine treatment failures in vivo in Gabonese children. Malar J.

[12] Ndounga M, Tahar R, Basco LK, Casimiro PN, Malonga DA, Ntoumi F (2007). Therapeutic efficacy of sulfadoxine–pyrimethamine and the prevalence of molecular markers of resistance in under 5-year olds in Brazzaville, Congo. Trop Med Int Health.

[13] Wang P, Lee CS, Bayounmi R, Djimde A, Doumbo O, Swedberg G (1997). Resistance to antifolates in *Plasmodium falciparum* monitored by sequence analysis of dihydropteroatesynthetase and dihydrofolatereductase alleles in a large number of field simples of diverse origins. Mol Biochem Parasitol.

[14] Faye B, Ndiaye M, Ndiaye JL, Annie A, Tine RC, Lo AC (2011). Prevalence of molecular markers of *Plasmodium falciparum* resistance to sulfadoxine-pyrimethamine during the intermittent preventive treatment in infants coupled with the expanded program immunization in Senegal. Parasitol Res.

[15] Henry M, Diallo I, Bordes J, Ka S, Pradines B, Diatta B (2006). Urban malaria in Dakar, Senegal: chemosusceptibility and genetic diversity of *Plasmodium falciparum* isolates. Am J Trop Med Hyg.

[16] Ndiaye D, Daily JP, Sarr O, Ndir O, Gaye O, Mboup S (2005). Mutations in *Plasmodium falciparum* dihydrofolate reductase and dihydropteroate synthase genes in Senegal. Trop Med Int Health.

[17] Ndiaye M, Tine R, Faye B, Ndiaye JL, Lo AC, Sylla K (2013). Selection of antimalarial drug resistance after intermittent preventive treatment of infants and children (IPTi/c) in Senegal. Am J Trop Med Hyg.

[18] Daniels R, Ndiaye D, Wall M, McKinney J, Séne PD, Sabeti PC (2012). Rapid, field-deployable method for genotyping and discovery of single-nucleotide polymorphisms associated with drug resistance in *Plasmodium falciparum*. Antimicrob Agents Chemother.

[19] Ndiaye D, Dieye B, Ndiaye YD, Van Tyne D, Daniels R, Bei AK (2013). Polymorphism in *dhfr*/*dhps* genes, parasite density and ex vivo response to pyrimethamine in *Plasmodium falciparum* malaria parasites in Thies, Senegal. Int J Parasitol Drugs Drug Resist.

[20] Rebaudet S, Bogreau H, Silaï R, Lepere JF, Bertaux L, Pradines B (2010). Genetic structure of *Plasmodium falciparum* and elimination of malaria, Comoros Archipelago. Emerg Infect Dis.

[21] Randrianarivelojosia M, Raherinjafy RH, Migliani R, Mercereau-Puijalon O, Ariey F, Bedja SA (2004). *Plasmodium falciparum* resistant to chloroquine and to pyrimethamine in Comoros. Parasite.

[22] Tall A, Rabarijaona LP, Robert V, Bedja SA, Ariey F, Randrianarivelojosia M (2007). Efficacy of artesunate plus amodiaquine, artesunate plus sulfadoxine-pyrimethamine, and chloroquine plus sulfadoxine-pyrimethamine in patients with uncomplicated *Plasmodium falciparum* in the Comoros Union. Acta Trop.

[23] Mouchet J, Carnevale P, Coosemans M, Julvez J, Manguin S, Richard-Lenoble D (2004). Biodiversité du paludisme dans le monde.

[24] Ndiaye D, Patel V, Demas A, LeRoux M, Ndir O, Mboup S (2010). A non-radioactive DAPI-based high-throughput in vitro assay to assess *Plasmodium falciparum* responsiveness to antimalarials-increased sensitivity of *P. falciparum* to chloroquine in Senegal. Am J Trop Med Hyg.

[25] Snounou G, Zhu X, Spiripoon N, Jarra W, Thaithong S, Brown KN (1999). Biased distribution of *msp1* and *msp2* allelic variant in *Plasmodium falciparum* populations in Thailand. Trans R Soc Trop Med Hyg.

[26] Parola P, Pradines B, Simon F, Carlotti MP, Minodier P, Ranjeva MP (2007). Antimalarial drug susceptibility and point mutations associated with drug resistance in 248 *Plasmodium falciparum* isolates imported from Comoros to Marseille, France, 2004–2007. Am J Trop Med Hyg.

[27] Andriantsoanirina V, Ratsimbasoa A, Bouchier C, Jahevitra M, Rabearimanana S, Radrianjafy R (2009). *Plasmodium falciparum* drug resistance in Madagascar: facing the spread of unusual *pfdhfr* and *pfmdr*-*1* haplotypes and the decrease of dihydroartemisinin susceptibility. Antimicrob Agents Chemother.

[28] RPP. Revue de la Performance du Paludisme dans l’union des Comores, rapport non publié; 2011.

[29] Anglaret X (2001). Trimethoprim-sulfamethoxazole prophylaxis in sub-Saharan Africa. Lancet.

[30] Whitty CJ, Jaffar S (2002). *Plasmodium falciparum* cross resistance. Lancet.

[31] Talisuna AO, Nalunkuma-Kazibwe A, Langi P, Mutabingwa TK, Watkins WW, Van Marck E (2004). Two mutations in dihydrofolate reductase combined with one in the dihydropteroate synthase gene predict sulphadoxine-pyrimethamine parasitological failure in Uganda children with uncomplicated *falciparum* malaria. Infect Genet Evol.

[32] McCollum AM, Schneider KA, Griffing SM, Zhou Z, Kariuki S, ter-Kuile F (2012). Differences in selective pressure on *dhps* and *dhfr* drug resistant mutations in western Kenya. Malar J..

[33] World Health Organization (2009). Report of the technical consultation on intermittent preventive treatment in infants (IPTi), technical expert group on preventive chemotherapy.

[34] WHO (2010). Policy recommendation on Intermittent Preventive Treatment during infancy with sulphadoxine-pyrimethamine (SP-IPTi) for Plasmodium falciparum Malaria control in Africa.

