# Supplementary material for: RDTs as a source of DNA to study Plasmodium falciparum drug resistance in isolates from Senegal and the Comoros Islands
Source: Malar J. 2015 Sep 29;14:373. doi: 10.1186/s12936-015-0861-6 (PMC4587814; doi:10.1186/s12936-015-0861-6)
Supplement: Additional file 1: — Table S1. Prevalence of dhfr and dhps alleles in Comoros over time. Prevalences in dhfr and dhps mutations were compared from Rebaudet et al. in 2010 and this study in 2012–2013. [file 12936_2015_861_MOESM1_ESM.docx]

|  |  |  | |  |  |  |
| --- | --- | --- | --- | --- | --- | --- |
| **Allele** | **Grande-Comore**  **2007** | | **Grande-Comore**  **2012-2013 RDT** | | | **Z-test** |
| ***dhfr*** | *Rebaudet* *et al* 2010 | | This study | |  |  |
| S108N | (13/26) 50% | | (115/123) 93% | |  | ***p<0.05*** |
| S108 and S108N Mix | (4/26)15.4% | | (4/123) 3.25% | |  | ***p=0.01*** |
| S108 WT | (9/26) 34.6% | | (4/123) 3.25% | |  | ***p<0.05*** |
| C59R | (13/26) 50% | | (67/96) 69.76% | |  | p=0.06 |
| C59 and C59R Mix | (2/26) 7.7% | | (27/96) 28% | |  | ***p=0.03*** |
| C59 WT | (11/26) 42.3% | | (2/96) 2.1% | |  | ***p<0.05*** |
| N51I | (10/26) 38.5% | | (60/96) 62.5% | |  | ***p=0.02*** |
| N51 and N51I Mix | (3/26) 11.5% | | (27/96) 28.1% | |  | p=0.08 |
| N51 WT | (13/26) 50% | | (9/96) 9.3% | |  | ***p<0.05*** |
|  |  | |  | |  |  |
| S108N and C59R Mix | (15/26) 57.7% | | (93/96) 96.9% | |  | ***p<0.05*** |
| S108N/C59R/N51I Mix | (13/26) 50% | | (86/96) 89.6 | |  | ***p<0.05*** |
| ***dhps*** |  | |  | |  |  |
| A437G | (1/25) 4% | | (37/124) 29.8% | |  | ***p=0.006*** |
| A437 and A437G | (0/25) 0% | | (5/124) 4% | |  | p=0.3 |
| A437 | (24/25) 96% | | (82/124) 66.1% | |  | ***p=0.002*** |
| K540E | (0/25) 0% | | (0/123) 0% | |  | … |
| K540 and K540E | (1/25) 4% | | (0/123) 0% | |  | ***p=0.02*** |
| K540 | (24/25) 96% | | (123/123) 100% | |  | ***p=0.02*** |
|  |  | |  | |  |  |
| A437G and K540E | (0/25) 0% | | (0/116) 0% | |  | … |
|  |  | |  | |  |  |
| MOI | 1.39** | | 1.47*** | |  |  |

** microsatelite typing of 6 markers

****msp1* & *msp2* typing
